# Early Energy Deficit in Huntington Disease: Identification of a Plasma Biomarker Traceable during Disease Progression

**DOI:** 10.1371/journal.pone.0000647

**Published:** 2007-07-25

**Authors:** Fanny Mochel, Perrine Charles, François Seguin, Julie Barritault, Christiane Coussieu, Laurence Perin, Yves Le Bouc, Christiane Gervais, Guislaine Carcelain, Anne Vassault, Josué Feingold, Daniel Rabier, Alexandra Durr

**Affiliations:** 1 INSERM, Hôpital de la Salpêtrière, UMR 679, Paris, France; 2 Assistance Publique-Hôpitaux de Paris, Hôpital de la Salpêtrière, Département de génétique et cytogénétique, Paris, France; 3 INSERM, Faculté de médecine et de pharmacie and Hôpital La Milêtrie, Poitiers, France; 4 Assistance Publique-Hôpitaux de Paris, Hôpital de la Salpêtrière, Laboratoire d'endocrinologie, Paris, France; 5 Assistance Publique-Hôpitaux de Paris, Hôpital d'enfants Armand Trousseau, Explorations fonctionnelles endocriniennes, Paris, France; 6 Assistance Publique-Hôpitaux de Paris, Hôpital de la Salpêtrière, Service de diététique, Paris, France; 7 Assistance Publique-Hôpitaux de Paris, Hôpital de la Salpêtrière, Laboratoire d'immunologie, Paris, France; 8 Assistance Publique-Hôpitaux de Paris, Hôpital Necker-Enfants malades, Laboratoire de biochimie métabolique, Paris, France; University of Giessen, Germany

## Abstract

Huntington disease (HD) is a fatal neurodegenerative disorder, with no effective treatment. The pathogenic mechanisms underlying HD have not been elucidated, but weight loss, associated with chorea and cognitive decline, is a characteristic feature of the disease that is accessible to investigation. We, therefore, performed a multiparametric study exploring body weight and the mechanisms of its loss in 32 presymptomatic carriers and HD patients in the early stages of the disease, compared to 21 controls. We combined this study with a multivariate statistical analysis of plasma components quantified by proton nuclear magnetic resonance (^1^H NMR) spectroscopy. We report evidence of an early hypermetabolic state in HD. Weight loss was observed in the HD group even in presymptomatic carriers, although their caloric intake was higher than that of controls. Inflammatory processes and primary hormonal dysfunction were excluded. ^1^H NMR spectroscopy on plasma did, however, distinguish HD patients at different stages of the disease and presymptomatic carriers from controls. This distinction was attributable to low levels of the branched chain amino acids (BCAA), valine, leucine and isoleucine. BCAA levels were correlated with weight loss and, importantly, with disease progression and abnormal triplet repeat expansion size in the HD1 gene. Levels of IGF1, which is regulated by BCAA, were also significantly lower in the HD group. Therefore, early weight loss in HD is associated with a systemic metabolic defect, and BCAA levels may be used as a biomarker, indicative of disease onset and early progression. The decreased plasma levels of BCAA may correspond to a critical need for Krebs cycle energy substrates in the brain that increased metabolism in the periphery is trying to provide.

## Introduction

Huntington disease (HD) is a neurodegenerative disease with autosomal dominant inheritance. The causal mutation is an abnormally expanded CAG trinucleotide repeat in the first exon of the HD1 gene on chromosome 4p, encoding a polyglutamine stretch in the huntingtin protein. Although transcriptional modifications, excitotoxicity, protein aggregation and loss of normal function of huntingtin, have been hypothesized to be responsible for the symptoms in patients [1], HD remains a devastating disorder with no effective treatment. Access to predictive DNA testing provides an opportunity for early therapeutic intervention in presymptomatic individuals.

Despite the ubiquitous expression of mutated huntingtin, only the pathophysiology of brain-related symptoms, which are relatively inaccessible to investigation, have received attention. However, unlike other neurodegenerative disorders, affected HD patients are known to lose weight [2], despite normal, or even increased, food intake [3], [4]. In a group of 361 HD patients, lower body mass index (BMI) has also been shown not to correlate with dystonia or with chorea [5]. The demonstration that mechanisms underlying weight loss in HD are related to disease onset would indicate that a more systemic, and possibly treatable, defect may be implicated early in the pathophysiology of HD.

Therefore, we conducted a thorough multiparametric investigation exploring the underlying causes of weight loss in a group of patients with no or little signs of the disease, compared to controls. This included the use of a multivariate statistical analysis of plasma components quantified by proton nuclear magnetic resonance (^1^H NMR) spectroscopy. We observed a systemic metabolic defect involving branched chain amino acids (BCAA) in HD, associated with early weight loss. The role of BCAA in mitochondrial intermediary metabolism may indicate a systemic attempt at compensating for the early energy deficit in HD. These results further support recent data relating altered mitochondrial energy metabolism in HD with defect in the regulation of transcription caused by mutated huntingtin [6].

## Materials and Methods

### HD patients, presymptomatic carriers and controls

We included 32 individuals with abnormal CAG repeats expansions (>36) in the HD1 gene (19 women and 13 men, mean age of 42±11 years, ranging from 28 to 80) and 21 controls (13 women and 8 men, mean age of 37±9.5 years, ranging from 27 to 62). In the HD group, 3 subgroups were constituted on the basis of their symptoms determined by the Unified Huntington disease rating scale (UHDRS) score [7]. There were 15 presymptomatic individuals who had previously applied for predictive testing, carriers of the HD1 mutation but without any significant motor or cognitive signs of HD, as defined by UHDRS≤3 (UHDRS 0.7±1.2). Ten HD mutation carriers were very mildly affected, with 4≤UHDRS≤25 (UHDRS 11.9±4.9), and were designated as early HD patients. The remaining 7 HD patients had moderate signs of the disease, with 26≤UHDRS≤70 (UHDRS 44.4±14.1), and were designated as mild HD patients. Since diagnostic and predictive testing in HD is highly confidential, family members were not chosen as a control group, which consisted of healthy volunteers unrelated to HD mutation carriers. All participants were enrolled in a clinical protocol promoted by the Assistance Publique des Hôpitaux de Paris (CRC 05129), and approved by the local ethics committee. Informed consent was obtained for all participants. Motor dysfunction was evaluated with the UHDRS and a total functional capacity score, TFC [8]. We used a simplified validated questionnaire – the patient health questionnaire, PHQ-9 – to evaluate depression [9]. Treatment and smoking habits were recorded. A detailed medical history was obtained for each participant, especially conditions known to affect body weight, such as malabsorption, renal insufficiency, endocrine dysfunction and inflammatory process (auto-immunity, cancer).

### Determination of weight balance and caloric intake

Height and weight were recorded the day of the clinical examination. The body mass index (BMI) was obtained by dividing weight (in kilograms) by height (in meters) squared. Bioelectrical impedance (Tanita®) was measured to evaluate the lean mass and fat mass of all participants. Since this was a cross-sectional study, changes in body weight were not evaluated prospectively. Instead, the kinetics of body weight was determined by subtracting current weight from the weight recorded in medical records of each participant 5 years before inclusion in the study.

To determine caloric intake accurately, both opened and closed-ended questionnaires were used. The opened-ended questionnaire consisted of a semi-quantitative recording of the patients' regular food and beverage consumption during the three days preceding the consultation. The accuracy of this questionnaire was verified one month later with a closed-ended questionnaire that was sent to the homes of all participants. A series of diagrams was used, reflecting the amount of each class of food and beverage consumed over a 24-hour period. The same methodology was strictly applied to assess caloric intake in HD patients, presymptomatic carriers and controls. In order to interpret changes in body weight, major changes in eating habits during the past 5 years were also recorded, especially diets intended to gain or lose weight. Mean total calories, proteins, lipids and sugar intake for both the HD and control groups were calculated using an automated system (Diaeta software®).

### Multiparametric evaluation of weight balance

A standardized protocol was designed to thoroughly evaluate all possible causes of weight loss and to avoid biases related to food intake and circadian fluctuations. It included sequentially: (i) a minimal 12-hour fast the night preceding the examination, (ii) collection of blood and urine at the same time (9 am), (iii) ingestion of a standardized meal (450 calories) over a period of 10 minutes, (iv) collection of a second blood sample to determine ghrelin values 1 hour after eating. Samples were stored on ice for immediate analyses or frozen at −80°C for further analyses.

Routine analyses of blood chemistry, including liver and kidney function, were performed. Serum insulin growth factor type 1 (IGF1) concentrations were measured using a specific immunoradiometric assay (IGF1 RIACT, Cis-Bio International, Gif- sur- Yvette, France). The three main axes involved in the regulation of weight balance were explored: (i) inflammation, by measuring of CRP, ESR, IL1β and IL6 by ELISA (Diaclone, Besançon, France), (ii) endocrine function, by measuring (at 9 am), fasting serum cortisol, insulin, tetraiodothyronine (T4L) and thyroid stimulating hormone (TSH) levels, fasting and fed ghrelin (8089-K, Linco Research, St Louis, USA) levels, and fasting leptin levels (HL-81HK, Linco Research, Paris, France); (iii) intermediary metabolism, by measuring plasma amino acids and acylcarnitines, as well as urinary organic acids [10], [11].

For comparison of means, ANOVA or non-parametric tests were used as appropriate (SPSS® software).

### ^1^H NMR spectroscopy of plasma

Plasma samples were prepared for ^1^H NMR spectroscopy with minimal handling as described [12]. For statistical analyses of the whole metabolic profile, spectra were data reduced in numerical format by integrating spectral regions (buckets) every 0.02 ppm (parts per million) and scaled to the total intensity of the spectrum with Amix 3.6.8 software (Bruker Analytische Messtechnik, Germany) from 0.8 to 8.6 ppm; the water peak area was excluded from each spectrum (4.4 to 5.2 ppm). Accordingly, each bucket of the NMR spectrum corresponded to a single X variable. Metabonomic studies, e.g. partial least squares analysis (PLS), consist in multivariate statistical analyses with as many components as variables. PLS (Simca-P® 11.0 software, Umetrics, Sweden) best describe the variation within the NMR spectra according to a priori classification, corresponding to a Y variable, which was the UHDRS score in our study. Therefore, the pair-wise PLS scores were plotted to identify principal components, maximizing the covariance between all X (NMR spectrum) and Y (UHDRS) variables. The greatest dispersion of the spectral profiles is usually observed in the two first components of the analyses. A contribution plot was then analyzed in order to determine the respective weight of variables that contributed most to the difference between groups.

## Results

### Clinical characteristics of the HD cohort

The 3 groups defined according to UHDRS scores showed similar ages at examination (Table 1). Abnormal CAG repeats in the mildly affected group were slightly smaller than in the early group, but the latter were older at examination (Table 1). Early affected HD patients had low chorea subscores (4.8±3.7), and all mildly affected patients were autonomous and were able to live on their own, as shown by mean TFC scores above 10 (Table 1). Depression scores of the HD group were higher than those of controls (7.3±6.6, n = 32 versus 0.7±1.6, n = 21, p<0.001). Smoking was also more prevalent in the HD group (n = 12/32 versus 1/21 in controls, p = 0.006).

**Table 1:** Clinical characteristics of Huntington disease cohort.

	Presymptomatic	Early HD	Mild HD	***p***
	**UHDRS = 0.7±1.2 (0–3)**	**UHDRS = 11.9±4.9 (7–21)**	**UHDRS = 44.4±14.1 (30–64)**	
	**n = 15**	**n = 10**	**n = 7**	
**CAG repeat expansion:** (Number of repeats)	42±2.0 (39–46)	45.8±4.7 (41–58)	44.4±2.0 (42–48)	*0.017*
**Mean age at examination:** (Years)	41.4±12.9 (28–80)	38.2±9.3 (28–53)	49.3±6.0 (43–60)	*0.057*
**Depression score:** (30 maximal, worst)	4.4±4.5 (0–16)	10.4±8.5 (0–25)	9.3±5.5 (3–18)	*<0.001*
**UHDRS, Chorea subscore** *	0.2±0.6 (0–2)	4.8±3.7 (0–12)	16.9±5.8 (11–26)	
**UHDRS, Dystonia subscore** *	0	0	2.6±2.6 (0–7)	
**Total functional score:** (13 maximal, best)	13±0 (13)	12.1±0.9 (11–13)	10.3±2.2 (8–13)	*<0.001*

* Chorea subscore 28 maximal, worst, dystonia subscore 20 maximal, worst

The values represent the mean±standard deviations. Ranges are in parentheses. Comparisons of means (ANOVA) were made among the 3 HD groups. UHDRS  =  Unified Huntington disease rating scale, 120 is the maximal score of severity, up to 4 is considered as normal. The HD group included 15 strictly presymptomatic carriers, 10 patients at an early stage of the disease and 7 mildly affected patients. Ages at examination did not differ significantly among groups; presymptomatic HD carriers were not younger than affected patients. Total functional score was close to maximal in all groups, even in mildly affected patients, in whom the mean TFC was greater than 10. However, the depression score was pathological, even in presymptomatic carriers, and worsened with disease progression.

### Evidence of an early hypermetabolic state in HD

Retrospective analysis of body weight during the 5 years preceding the study showed a significant weight loss in the HD group compared to controls (p<0.001). The difference remained significant when men (p = 0.002) and women (p = 0.003) were analyzed separately (Table 2). Mean weight loss in early (−3.1±2.9 Kg) and mild HD patients (−5.0±7.1 Kg) was significantly greater than in presymptomatic carriers (−2.6±3.9 Kg) (p<0.01). Caloric intake recorded over the 3-day period and the 24-hour period was similar, so that the value, an essential determinant of weight balance, was considered to be reliable both in the HD group and in controls. No changes in diet or in conditions affecting body weight were recorded over the 5 years preceding the study either of these groups. Although HD mutation carriers had a higher caloric intake, their BMI was similar. In women, the BMI of the HD group was lower than the average for the French population (BMI = 23, in ‘Enquête décennale santé’, INSEE 2006), which was not reflected by the controls from our study (BMI = 21.3). Importantly, in HD men, BMI was significantly lower than in controls, and total caloric intake was inversely correlated with weight (p = 0.029) and lean body mass (p = 0.004), which clearly indicated a hypermetabolic state. Moreover, in presymptomatic carriers, daily caloric intake was higher than in controls (2195±495 Cal, n = 15 versus 1665±305 Cal, n = 21, p<0.001), as well as protein intake (85±24 g, n = 15 versus 70±14 g, n = 21, p = 0.025). We found no correlation between depression scores and food intake or between smoking habits and weight loss.

**Table 2:** Nutritional characteristics of patients with Huntington disease according to sex, compared to controls.

	Total			Men			Women		
	HD **(n = 32)**	Controls **(n = 21)**	***p***	HD **(n = 13)**	Controls **(n = 8)**	***p***	HD **(n = 19)**	Controls **(n = 13)**	***p***
**Weight loss (Kg) ** A	−3.3±4.5 (−13 to 6)	2.8±3.9 (−3 to 10)	*<0.001*	−2.8±4.6 (−13 to 2)	5.2±4.1 (0–10)	*0.002*	−3.6±4.6 (−12 to 6)	1.5±3.3 (−3 to 10)	*0.003*
**Present weight (kg)**	64.4±10.0 (48–89)	70.1±17.7 (49–112)	*0.140*	71.05±7.6 (61–88)	87.9±15.4 (66–112)	*0.003*	59.85±9.0 (48–78)	59.2±6.8 (49–68)	*0.826*
**Height (cm)**	168.7±8.1 (151–185)	170.3±6.6 (160–183)	*0.442*	175.7±5.7 (165–185)	176.2±4.7 (171–183)	*0.845*	163.8±5.6 (151–177)	166.7±4.7 (160–175)	*0.140*
**BMI (kg/cm^2^)**	22.6±3.0 (18–29)	24.0±5.0 (18–38)	*0.217*	23.05±2.8 (18–29)	28.35±5.3 (21–38)	*0.008*	22.3±3.1 (18–29)	21.3±2.1 (18–26)	*0.313*
**Lean Mass (kg) ** B	50.7±8.3 (39–68)	52.7±11.5 (39–72)	*0.466*	58.9±4.6 (53–68)	66.2±4.9 (57–72)	*0.003*	44.4±3.4 (39–51)	44.4±3.1 (39–48)	*0.996*
**Fat Mass (kg) ** B	20.9±.8 (11–37)	24.2±6.4 (13–38)	*0.124*	12.2±4.8 (7–25)	21.65±11.6 (9–43)	*0.017*	14.9±7.2 (6–29)	14.8±4.5 (9–22)	*0.989*
**Calories (/24 h) ** C	2020±530 (985–3040)	1665±305 (1280–2235)	*0.008*	2250±376 (1720–2960)	1760±355 (1280–2235)	*0.017*	1890±565 (983–3041)	1610±265 (1330–2130)	*0.103*
**Proteins (gr/24 h) ** C	81.3±23.7 (42–121)	70.0±14.1 (50–102)	*0.054*	86.3±23.3 (53–121)	78.6±15.9 (62–102)	*0.583*	78.4±24.1 (42–117)	64.6±10.0 (50–86)	*0.058*
**Lipids (gr/24 h) ** C	86.5±18.3 (53–120)	65.7±18.0 (47–126)	*<0.001*	90.4±15.1 (61–114)	68.9±11.9 (50–90)	*0.006*	84.2±19.9 (53–120)	62.4±21.2 (47–126)	*0.008*
**Sugar (gr/24 h) ** C	216.0±77.1 (76–375)	191.3±48.6 (123–310)	*0.201*	257.5±60.2 (186–375)	196.0±68.4 (123–310)	*0.071*	192.0±76.8 (76–372)	188.4±34.4 (130–256)	*0.846*

The values represent the mean±standard deviations. Ranges are in parentheses. The HD group was compared to controls (ANOVA).

A Determined retrospectively for a 5-year period.

B Determined by bioelectric impedance.

C Determined from a 3-day and a 24-hour questionnaire performed at a one-month interval. Weight loss was significant in the HD group, in both men and women. Despite significantly higher calories intake, carriers of huntingin mutations did not have a higher BMI (body mass index). In men, BMI and lean body mass were lower in the HD group despite higher food intake. These nutritional analyses demonstrate the existence of a hypermetabolic state in the early stages of HD.

ERS, CRP, serum interleukins IL 1ß (0.96±3.82 pg/ml versus 0.28±0.77 pg/ml) and IL 6 (0.54±2.50 pg/ml versus 0.39±0.89 pg/ml), on the one hand, and serum fasting cortisol (13.4±4.4 µg/100 ml versus 16.7±6.8 µg/100 ml), insulin (5.5±2.2 µU/ml versus 6.0±3.6 µU/ml), T4L (14.7±2.2 pmol/l versus 16.4±1.9 pmol/l) and TSH (1.92±0.90 mUI/l versus 1.80±0.65 mUI/l), on the other hand, were similar in the HD group and controls. Therefore, common causes of hypercatabolic state, i.e. inflammation and classical endocrine dysfunctions, were excluded. There was no glycosuria, and fasting blood glucose levels were in the normal range in the HD group (4.7±0.4 mmol/l).

### Identification of a systemic metabolic defect by plasma ^1^H NMR

**Figure 1:**
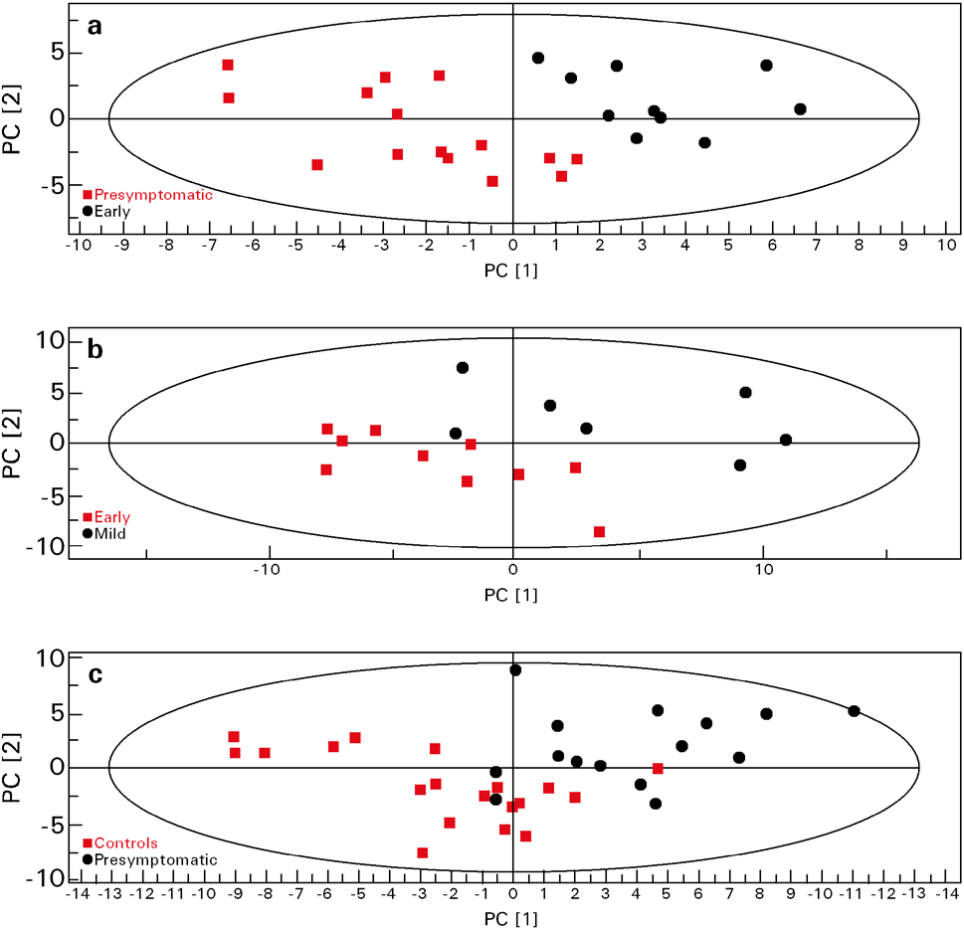
Partial least square (PLS) analyses of NMR spectra of plasma samples from HD patients with no or little signs of the disease and controls. Three groups of presymptomatic, early and mildly affected HD patients were constituted on the basis of their UHDRS scores, as described in the methods. The first and second components in the X space (NMR spectrum) are denoted PC[1] and PC[2] respectively. PLS score plots (PC[1]/PC[2]) of pair-wise compared groups show the greater variation within the NMR spectrum according to a priori classification with UHDRS. There is a clear separation between presymptomatic and early HD patients (a), as well as between early and mildly affected HD patients (b). Despite some overlap, presymptomatic mutation carriers can also be distinguished from controls (c).

PLS analyses distinguished HD individuals at different stages of the disease as shown in Figure 1. The difference between presymptomatic carriers and early HD is shown in Figure 1a. Early and mildly affected patients could be distinguished (Figure 1b), as could controls and presymptomatic carriers, despite some overlap (Figure 1c).

**Figure 2:**
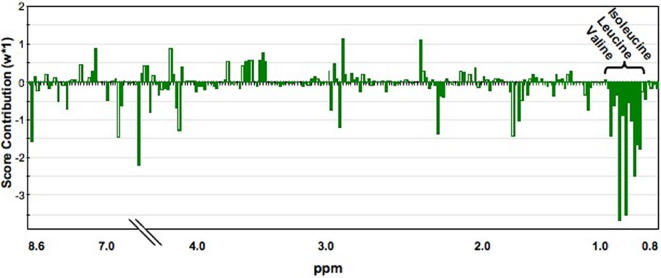
Differences in the relative concentrations of branched chain amino acids in plasma are responsible for the differences among the HD groups. PLS-contribution plots allow comparison of plasma metabolic profiles in early affected HD patients and presymptomatic carriers. NMR variables that have the greatest weight (w*_1_; scaled in units of standard deviation), therefore contributing most to the separation between HD groups, are decreased concentrations (>2SD) of metabolites located between 0.85 and 1.0 ppm: valine, leucine and isoleucine. A similar contribution plot was obtained when plasma metabolic profiles from mildly affected HD patients were compared to early HD patients (data not shown).

Metabolic profiles of plasma from early affected HD patients were then compared to those of presymptomatic carriers, those of mildly affected HD patients to early HD patients. The spectral region that contributed to differences among the HD groups determined from PLS contribution plots is shown in Figure 2. There was a significant (>2SD) decrease along with disease progression in the plasma concentrations of a group of variables from the buckets located between 0.85 to 1.0 ppm on the NMR spectrum. These peaks correspond to the branched chain amino acids (BCAA), valine, leucine and isoleucine. No other significant differences among the groups were detected in the spectra even though very small buckets (0.02 ppm) were analyzed.

### Confirmation that plasma BCAA are low in HD

**Figure 3:**
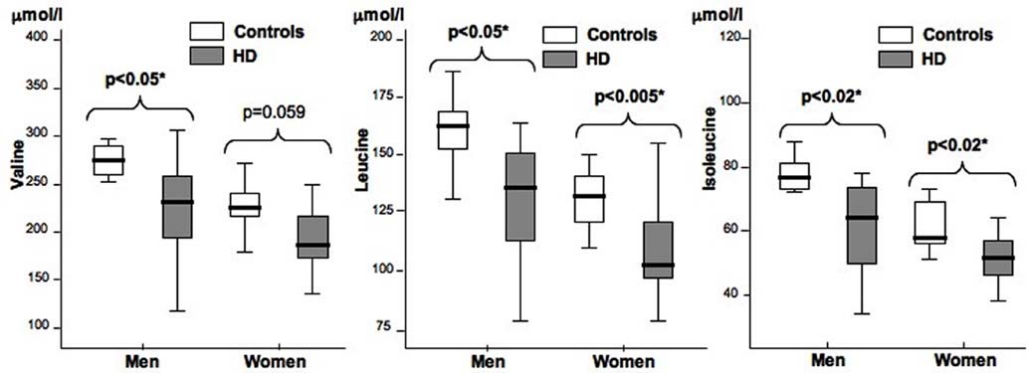
The levels of branched chain amino acids are significantly different in HD patients and controls. The concentrations of valine, leucine and isoleucine in plasma were determined by ion exchange chromatography. Comparisons of means (ANOVA) were made between men or women with HD and their respective controls. In men, there was a significant decrease of valine, leucine and isoleucine in the HD group. In women, similar results are observed for leucine and isoleucine.

To confirm that BCAA are affected in HD, we also measured their concentrations in plasma by ion exchange chromatography. Valine, leucine and isoleucine levels were significantly lower in the HD group compared to controls (209±47 µmol/l versus 245±44 µmol/l, p = 0.009; 118±24 µmol/l versus 144±23 µmol/l, p<0.001 and 56±12 µmol/l versus 68±15 µmol/l, p = 0.002, respectively). In addition, the levels of each BCAA were correlated with the observed weight loss in the patients (p = 0.007, 0.003 and 0.017), and, more importantly, negatively correlated with UHDRS values (p = 0.017, <0.0001 and 0.003) in both men (p = 0.035, 0.019 and 0.036) and women (p = 0.007 for leucine and 0.01 for isoleucine). The number of CAG repeats was also negatively correlated with valine (p = 0.015), leucine (p = 0.018) and isoleucine (p = 0.020). Although the BMI values of women with HD were similar to those of controls, they had significantly lower leucine (109±19 µmol/l versus 134±22 µmol/l, p = 0.002) and isoleucine (53±10 µmol/l versus 65±16 µmol/l, p = 0.014) levels (Figure 3). Interestingly, the plasma levels of the three BCAA were significantly lower in patients at an early stage of the disease compared to presymptomatic carriers (188±35 µmol/l versus 229±50 µmol/, p = 0.042, 105±21 µmol/l versus 130±24 µmol/l, p = 0.019 and 49±9 µmol/l versus 62±12 µmol/l, p = 0.024). The plasma levels of valine (228±50 µmol/l versus 245±44 µmol/l), leucine (130±24 µmol/l versus 144±23 µmol/l) and isoleucine (62±12 µmol/l versus 68±15 µmol/l) tended to be lower in presymptomatic carriers than controls, although the difference was not significant. There was no correlation between BCAA levels and age or smoking habits.

All other metabolic markers were similar in the HD group and in controls. However, IGF1 levels were significantly lower in the HD group than in controls (175±48 ng/ml versus 210±47 ng/ml, p = 0.011) and negatively correlated with UHDRS scores (p = 0.004). IGF1 levels were also correlated with leucine (p = 0.04) and isoleucine (p = 0.02) levels. There was no correlation between IGF1 levels and parameters associated with weight (BMI, lean and fat body mass) or caloric intake.

In men, fasting and postprandial ghrelin levels were higher in the HD group than in controls (1797±719 pg/ml versus 1077±453 pg/ml, p = 0.024 and 1365±538 pg/ml versus 860±291 pg/ml, p = 0.021 respectively). However, women with HD and controls had similar BMI values. Accordingly, ghrelin levels were negatively correlated with BMI (p = 0.008) but not with the UHDRS score. Fasting ghrelin also correlated with caloric intake in men (p = 0.029), consonant with its orexigenic activity. Importantly, the ratio between pre- and post-prandial ghrelin levels were strictly identical in the HD group compared to controls (1.35 versus 1.36) indicating that the negative feedback on hypothalamus induced by food intake is functional in HD. Leptin levels, which vary independently but inversely to ghrelin, were lower in men with HD compared to controls (4.4±3.3 ng/ml versus 12.4±11.5 ng/ml, p = 0.028), but not in women, and were correlated with weight (p<0.001) and fat body mass (p<0.001), but not with the UHDRS score.

## Discussion

This study is the first comprehensive investigation of the physiopathology of weight loss in HD. We retrospectively confirmed that weight loss begins early in the disease [5], despite higher caloric intake, and is evident even in presymptomatic mutation carriers and those with mild chorea, ruling out the role of hyperactivity. This hypermetabolic state is not explained by common mechanisms like inflammation or altered endocrine functions, both of which have been incriminated in the pathophysiology of HD [13], [14]. High ghrelin levels and low leptin levels in men with HD were reflective of their low BMI compared to controls, and did not evolve with the disease. Thus the ability of the hypothalamus to inhibit ghrelin synthesis after food intake is functional in HD, contrary to a previous report [15]. Therefore, the hypermetabolic state of HD patients seems to be part of the pathological process induced by mutated huntingtin.

Our ^1^H NMR spectroscopic analyses showed that a systemic metabolic defect is associated with the status of HD carriers, as well as disease progression. A selective decrease in branched chain amino acids plasma concentrations distinguished presymptomatic carriers from patients at an early stage of the disease and patients at an early stage from mildly affected patients. Decreased plasma BCAA levels were previously documented in more severely affected HD patients, but the authors did not evaluate the patients' diet or weight [16], [17]. Recently, a decrease of plasma BCAA was found by mass spectrometry in HD patients at different stages of the disease, in association with various metabolic changes [18]. Our metabolic screening was sufficiently detailed to demonstrate that plasma BCAA are the only metabolites that differ between the HD patients and controls. A significant difference between patients at an early stage of the disease and presymptomatic carriers, was also observed regardless of sex. The difference between presymptomatic carriers and controls, did not reach statistical significance, however, consistent with the partial overlap between the metabolic profiles of plasma observed by ^1^H NMR spectroscopy. This is most likely due to the heterogeneity of the presymptomatic group in which the estimated time to disease onset varies among individuals, so that the metabolic profiles of some presymptomatic carriers can be similar to those of controls. Interestingly, the decrease in the levels of BCAA correlated with weight loss in HD patients. Most importantly, it varied with HD itself, i.e. with the UHDRS scores of the patients and the number of CAG repeats in their mutations. Therefore, plasma BCAA can be considered as relevant biomarkers of the early stages of HD. For more severely affected patients, further analyses should be performed. It is of note that very few metabonomic studies have led to the identification of biomarkers that can be used routinely for the follow up of patients. This is due to common experimental and analytical biases in humans that include dietary intake, time of sample collection, sample conditioning and chemical shifts due to changes in pH [19], [20]. The rigorous control of these parameters in our study likely explains the accuracy of our NMR findings.

The implication of BCAA in mitochondrial intermediary metabolism, both in brain and peripheral tissues, further supports an important role for energy deficit in HD. A reduction in ATP production was shown in brain of HD mice, including presymptomatic mice [21]. A significant reduction in ATP levels and mitochondrial respiration was also evidenced in striatal cells of HD mice, although the respiratory chain complexes were not impaired [22]. In HD patients, there is strong evidence for hypometabolism in the brain where glucose consumption is reduced, especially in the basal ganglia, even in presymptomatic mutation carriers [23], [24], [25]. The underlying cause of this early energy deficit in HD brain is currently unknown, but impaired glycolysis [26], citric acid cycle [27] and/or oxidative phosphorylation [22] may be involved. Recently, mutated huntingtin was shown to decrease the expression of PGC-1α (peroxisome proliferators-activated receptor gamma coactivator-1α) in the striatum of HD mice and patients, through a CREB-dependent transcriptional inhibition [28]. PGC-1α is a transcriptional coactivator that regulates key energetic metabolic pathways, both in the brain and peripheral tissues [29]. The possible role of PGC-1α in HD was initially suspected from the observation of selective striatal lesions in the PGC-1α knockout mouse [30]. Down-regulation of PGC-1α in HD striatum was then shown to affect mitochondrial energy metabolism, possibly by impairing oxidative phosphorylation [28]. In addition, the inhibition of succinate dehydrogenase, by 3-nitropropionic acid or malonate, mimics HD neuropathology in mice [31], indicating that a lack of substrates for the citric acid cycle and the respiratory chain is implicated in the energy deficit in HD brain. Importantly, mitochondrial oxidation of BCAA leads to the production of acetyl-CoA and succinyl-CoA, two key intermediates of the citric acid cycle. Insufficient caloric or protein intake was excluded in our study, as well as impairment of the BCAA oxidation pathway since organic acid levels in urine were normal. Therefore, the decrease in plasma BCAA observed in the HD group probably results from the activation of a compensatory mechanism to provide energy substrates to the citric acid cycle, as described in various cachexia-producing illnesses [32], [33]. The correlation between decreased BCAA levels and weight loss in our study suggests that excessive mobilization and oxidation of BCAA to produce energy in muscle is associated with weight loss and reduced lean body mass. The observation of weight loss prior to neurocognitive decline suggests that neurological symptoms are exacerbated when substrates from the periphery become insufficient to compensate for the energy deficit in the HD brain. The normal rate of oxygen consumption recently observed after striatal infusion of succinate [34] supports the idea that providing energy through an increase in both systemic and cerebral citric acid cycle intermediates may be a promising therapeutic approach in HD.

BCAT (branched-chain aminotransferase) is the first enzyme involved in BCAA catabolism and is preferentially expressed in muscle. Since altered energy metabolism has been shown in muscle of HD patients [35], it remains to be determined whether the systemic decrease in plasma BCAA also reflects an adaptive response to a peripheral energy deficit in HD [36], in addition to the energy deficit in the brain. As the two key enzymes of BCAA oxidation, BCAT and BCKDH (α-keto acid dehydrogenase), are widely expressed in the brain [37], the decrease in BCAA levels detected in the cerebrospinal fluid of HD patients [16] may indicate that BCAA oxidation is also activated in the brain to compensate for the local energy deficit. Indeed, BCAA play an important role in brain metabolism, accounting for up to 20% of whole-body BCAA metabolism [38]. Leucine, in particular, is a nitrogen donor for the glutamate-glutamine shuttle, with transamination of α-keto-isocaproate to leucine leading to the conversion of glutamate into α-ketoglutarate [39]. Low levels of BCAA in the brain are, therefore, likely to impair the buffering of glutamate, in accordance with previous studies showing altered glutamate-glutamine cycling in HD [40]. Recently, excessive oxidation of BCAA was observed in a BCKDH kinase knockout mouse, emphasizing the importance of BCAA in intermediary metabolism, in both brain and peripheral organs [41]. Indeed, the systemic decrease in BCAA levels was associated not only with weight loss, but also with motor dysfunction in adult mice.

Finally, our study showed that low plasma BCAA levels are associated with reduced IGF1 levels in the HD group despite a higher protein and caloric intake than controls. The tight connection between IGF1 and essential amino acids has been repeatedly demonstrated [42], [43], [44]. Interestingly, huntingtin is a substrate of the serine-threonine Akt pathway, which is activated by IGF1 [45]. Reduced activation of the Akt pathway has been shown to decrease phosphorylation of mutated huntingtin, resulting in increased neuronal toxicity [46]. Low levels of IGF1 in HD patients might contribute to the alteration in Akt activation observed in HD animal models and patients [47].

This study provides the first demonstration of a systemic metabolic defect in HD, associated with early weight loss. Plasma BCAA represent a promising and accessible biomarker that may be useful for the detection of disease onset and for monitoring therapeutic trials in patients at an early stage of the disease. Decreased plasma BCAA levels in HD strongly suggest a critical need in the brain for citric acid cycle substrates provided by peripheral organ metabolism.
